# Peripheral Blood and Cerebrospinal Fluid Cytokine Levels in Guillain Barré Syndrome: A Systematic Review and Meta-Analysis

**DOI:** 10.3389/fnins.2019.00717

**Published:** 2019-07-16

**Authors:** Ting Sun, Xi Chen, Sha Shi, Qingshan Liu, Yong Cheng

**Affiliations:** Key Laboratory of Ethnomedicine for Ministry of Education, Center on Translational Neuroscience, College of Life and Environmental Sciences, Minzu University of China, Beijing, China

**Keywords:** inflammation, cytokine, Guillain Barré Syndrome, peripheral blood, cerebrospinal fluid, meta-analysis

## Abstract

**Background:** Guillain Barré Syndrome (GBS) is an autoimmune disorder caused by the immune-mediated damage of the peripheral nervous system. Increasing evidence suggests that inflammatory cytokines are important mediators for the onset and progression of GBS. A number of clinical studies have demonstrated elevated levels of T helper-1 (Th1-), Th2-, and Th17-related cytokines in patients with GBS; however, the results were inconsistent across studies.

**Methods:** We performed a systematic review and a meta-analysis of studies comparing the levels of inflammatory cytokines in the cerebrospinal fluid and peripheral blood between patients with GBS and healthy individuals, using Comprehensive Meta-Analysis Version 2 software. A database search identified 30 studies comprising 1,302 patients with GBS and 1,073 healthy controls.

**Results:** The random-effects meta-analysis demonstrated that peripheral blood tumor necrosis factor-α (Hedges g, 1.544; 95% confidence interval (CI), 0.923–2.165; *p* < 0.001), interleukin-1β (IL-1β; Hedges g, 0.678; 95% CI, 0.183–1.172; *p* = 0.007), IL-6 (Hedges g, 0.630; 95% CI, 0.100–1.160; *p* = 0.02), IL-4 (Hedges g, 0.822; 95% CI, 0.220–1.423; *p* = 0.007), IL-17 (Hedges g, 1.452; 95% CI, 0.331–2.573; *p* = 0.011), interferon-γ (Hedges g, 1.104; 95% CI, 0.490–1.719; *p* < 0.001), and C-reactive protein (Hedges g, 0.909; 95% CI, 0.453–1.365; *p* < 0.001) levels were significantly increased in patients with GBS when compared with healthy controls. Contrastingly, the blood IL-10 and transforming growth factor-β levels were not significantly associated with GBS. Furthermore, the meta-analysis found that cerebrospinal fluid IL-17 levels were significantly associated with GBS (Hedges g, 1.882; 95% CI, 0.104–3.661; *p* = 0.038).

**Conclusion:** Altogether, our results clarified the circulating inflammatory cytokine profile in patients with GBS, and revealed that Th1-, Th2-, and Th17-related cytokines were highly elevated in the GBS patients, suggesting the potential use of these cytokines as biomarkers for GBS.

## Introduction

Guillain Barré Syndrome (GBS) is an immune-mediated disorder of the peripheral nervous system characterized by muscle weakness (Eldar and Chapman, 2014). GBS has a number of subtypes, among which the most common subtype is the acute inflammatory demyelinating polyradiculoneuropathy, followed by acute motor axonal neuropathy (van den Berg et al., 2014). The disease prevalence has been reported to be one or two cases per 100,000 individuals every year, and most of the patients had an infection before the onset of the disease (van den Berg et al., 2014). Although it is generally believed that the post-infectious immune dysfunction-mediated demyelination of the peripheral nervous system is the cause of GBS, the precise etiology of the disease is still inadequately understood.

It has been proposed that a series of immune responses including macrophage and complement activation, and T-cell mediated cytotoxicity causes the demyelination of the peripheral nervous system and axonal damage leading to the onset of GBS (Hartung and Toyka, 1990; Wanschitz et al., 2003; Debnath et al., 2018b). It is well-known that the integrated actions of various cytokines play crucial roles in the differentiation and activation of immune cells such as B lymphocytes, T lymphocytes, and macrophages. Thus, cytokines are thought to be important mediators in the upstream and downstream processes of many inflammatory diseases. Indeed, various studies suggest that cytokines produced by various T-cell lineages including Th1, Th2, and Th17 play important roles in the onset and progression of GBS (Zhang et al., 2013; Li et al., 2014). Several studies demonstrated that the levels of inflammatory cytokines–tumor necrosis factor-α (TNF-α), interleukin-6 (IL-6), interferon-γ (IFN-γ), and IL-17–were elevated in the patients with GBS compared to healthy controls (Hohnoki et al., 1998; Ossege et al., 2000; Nyati et al., 2011; Han et al., 2014). However, other studies did not show significant differences between patients with GBS and controls for these cytokines (Exley et al., 1994; Press et al., 2001; Beppu et al., 2015), and moreover, one study showed that blood IL-17 levels were significantly down-regulated in the patients with GBS (Doncel-Perez et al., 2016). Therefore, a systematic review and meta-analysis is necessary to address the inconsistencies in available data.

In this study, we performed a systematic review of literature on cytokines in the cerebrospinal fluid (CSF) and blood of patients with GBS and compared the data with that of healthy controls, and pooled the individual cytokine data from included studies with a meta-analytic technique. Moreover, we used sub-group and meta-regression analyses to address the between-study heterogeneity found in this meta-analysis.

## Materials and Methods

This systematic review and meta-analysis was performed according to the PRISMA (Preferred Reporting Items for Systematic reviews and Meta-analysis) guidelines (Moher et al., 2009).

### Search Strategy and Study Selection

A systematic review of peer-reviewed English articles was performed by two independent investigators, and the PubMed and Web of Science databases were searched until November 2018. The search terms used in this systematic review were as follows: (inflammation or cytokine or chemokine or tumor necrosis factor or interleukin or interferon or C-reactive protein) and (GBS). We included studies with reported data on CSF and blood cytokine levels in patients with GBS and healthy, control individuals. The exclusion criteria were as follows: (1) *Ex vivo* data with reported cytokine concentrations; (2) data without a control group; (3) single case study, and (4) Cytokines were measured in less than three studies.

### Data Extraction

The data were extracted by one investigator and checked by another. We extracted data on sample size, mean cytokine concentration, standard deviation (SD), and *p*-value to calculate the effective size (ES). We also extracted age, gender, sample source, disease duration, disease severity, diagnosis, and medication status for potential moderator analysis (Supplementary Table 1).

### Data Analysis

The statistical analyses for the cytokine differences between cases and controls in the meta-analysis were performed using Comprehensive Meta-Analysis Version 2 software (Biostat Inc., Englewood, NJ, USA) (Du et al., 2018). The sample size, mean cytokine concentration, and SD were used to calculate the ES. If cytokine concentration data were not available, the ESs were generated by sample size and *p*-value. We calculated the ES as standardized mean differences (SMD) of cytokine concentrations between patients with GBS and healthy individuals, and converted the values to Hedges g, which adjusted the ES based on the sample size (Wei et al., 2018). Each blood or CSF cytokine analyzed in this study was subjected to meta-analysis for providing an ES estimate. The random-effects meta-analysis was chosen in this study because both between-study and within-study heterogeneities were postulated to influence the true ES (Qin et al., 2016). Moreover, we used a sensitivity analysis, by removing one study at a time, to determine whether the statistical significance was influenced by any single study.

The Cochrane's *Q*-test and I^2^ index were used to determine the between-study heterogeneity as described previously (Chen et al., 2018). It should be noted that for the Cochrane's *Q*-test, *p* < 0.1 was considered statistically significant; I^2^ index of 075, 0.50, and 0.25 denoted high, moderate, and small levels of heterogeneity, respectively. Furthermore, we used unrestricted maximum-likelihood random-effects meta-regressions of ES to assess whether age, gender (proportion of male), and publication year influenced the outcomes of the meta-analysis. Additionally, we evaluated publication bias using Egger's test which assesses the asymmetry of the funnel plot.

We considered *p* < 0.05 to be statistically significant except where noted.

## Results

**Figure 1:**
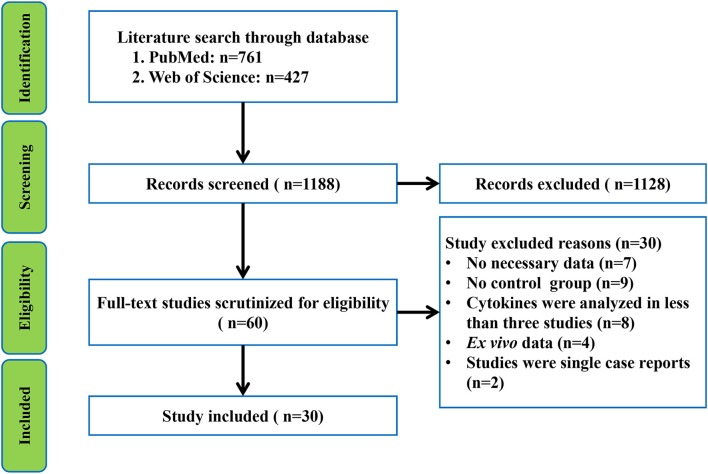
Preferred Reporting Items for Systematic reviews and Meta-analysis flowchart of the literature search.

The initial search retrieved 761 records from PubMed and 427 records from Web of Science. After screening the titles and abstracts of the 1,188 records, 60 relevant studies were selected for full text scrutiny. Thirty of the 60 studies were excluded after reading the full text because they lacked the necessary data (*n* = 7); lack of a control group (*n* = 9); reported *ex vivo* cytokine data (*n* = 4); single case reports (*n* = 2), and blood or CSF cytokines were analyzed in less than three studies (*n* = 8). Thus, a total of 30 studies comprising 1,302 patients with GBS and 1,073 healthy individuals were included in the meta-analysis (Sharief et al., 1993; Exley et al., 1994; Sindern et al., 1996; Creange et al., 1998; Hohnoki et al., 1998; Ossege et al., 2000; Press et al., 2001; Radhakrishnan et al., 2003, 2004; Deng et al., 2008; Nyati et al., 2010, 2011; Sainaghi et al., 2010; Li et al., 2012, 2013, 2014, 2018a,b; Liang et al., 2012; Wang et al., 2012; Han et al., 2014; Beppu et al., 2015; Du et al., 2015; Chang et al., 2016; Doncel-Perez et al., 2016; Zhang et al., 2016; Kharwar et al., 2017; Debnath et al., 2018a,b; Ethemoglu and Calik, 2018) (Flowchart see Figure 1).

### Main Associations of GBS With Blood Cytokines

**Figure 2:**
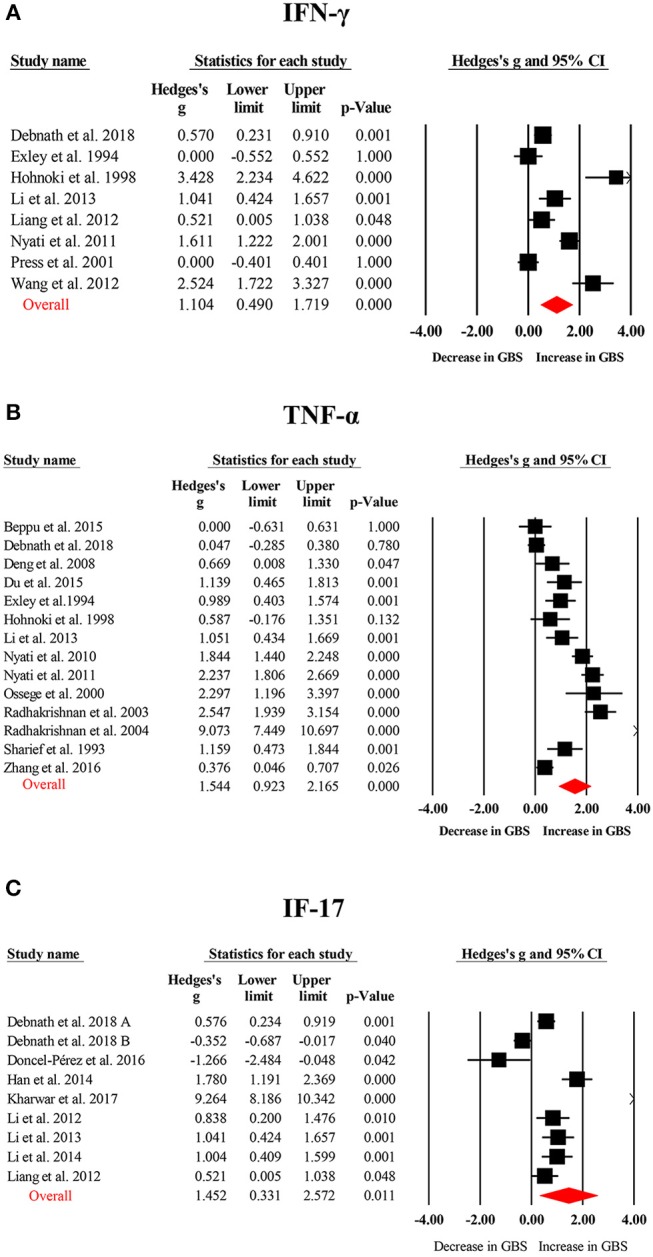
Studies of blood tumor necrosis factor-α (TNF-α), interferon-γ (IFN-γ), and interleukin-17 (IL-17) in Guillain Barré Syndrome. Forest plot displaying random-effects meta-analysis results of the association between IFN-γ **(A)**, TNF-α **(B)**, IL-17 **(C)** and Guillain Barré Syndrome. The sizes of the squares are proportional to study weights.

**Figure 3:**
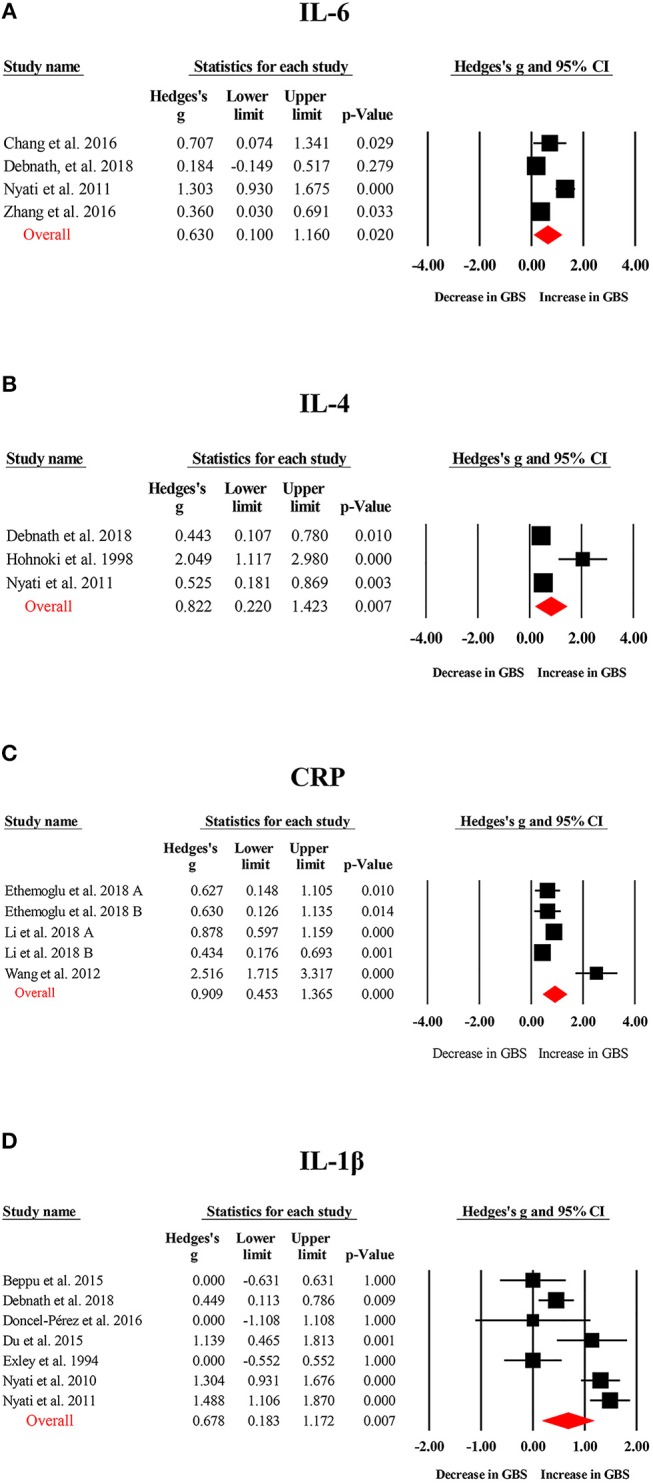
Studies of blood IL-1β, IL-4, IL-6, and CRP in Guillain Barré Syndrome. Forest plot displaying random-effects meta-analysis results of the association between IL-6 **(A)**, IL-4 **(B)**, CRP **(C)**, IL-1β **(D)** and Guillain Barré Syndrome. The sizes of the squares are proportional to study weights.

Random-effects meta-analysis showed that patients with GBS had significantly elevated TNF-α (Hedges g, 1.544; 95% confidence interval (CI), 0.923–2.165; *p* < 0.001), IL-1β (IL-1β; Hedges g, 0.678; 95% CI, 0.183–1.172; *p* = 0.007), IL-6 (Hedges g, 0.630; 95% CI, 0.100–1.160; *p* = 0.02), IL-4 (Hedges g, 0.822; 95% CI, 0.220–1.423; *p* = 0.007), IL-17 (Hedges g, 1.452; 95% CI, 0.331–2.572; *p* = 0.011), IFN-γ (Hedges g, 1.104; 95% CI, 0.490–1.719; *p* < 0.001), and C-reactive protein (CRP; Hedges g, 0.909; 95% CI, 0.453–1.365; *p* < 0.001) levels when compared with healthy controls (Figures 2, 3; Table 1). However, blood IL-10 and transforming growth factor- β (TGF-β) levels did not show significant differences between the cases and controls (Table 1).

**Table 1:** Summary of comparative outcomes for measurements of blood cytokine levels.

**Blood cytokine**	**No. of studies**	**No. with GBS/controls**	**Main effect**			**Heterogeneity**				**Publication bias**	
			**Hedges g (95% CI)**	***z* score**	***p* value**	**Q statistic**	***df***	***p* value**	**I^**2**^ statistic**	**Egger intercept**	***p* value**
TNF-α	14	479/382	1.544 (0.923–2.165)	4.873	< 0.001	230.279	13.000	< 0.001	94.335	5.922	0.053
CRP	5	304/315	0.909 (0.453–1.365)	3.910	< 0.001	25.468	4.000	< 0.001	84.294	4.152	0.216
IFN-γ	8	286/301	1.104 (0.490–1.719)	3.524	< 0.001	79.827	7.000	< 0.001	91.231	5.163	0.175
TGF-β	5	124/115	0.293 (−0.534–1.121)	0.694	0.487	29.287	4.000	< 0.001	86.342	3.297	0.319
IL-1β	7	200/207	0.678 (0.183–1.172)	2.687	0.007	40.784	6.000	< 0.001	85.288	−2.908	0.376
IL-4	3	144/153	0.822 (0.220 to1.423)	2.678	0.007	10.286	2.000	0.006	80.555	5.199	0.05
IL-6	4	244/216	0.630 (0.100–1.160)	2.331	0.020	21.740	3.000	< 0.001	86.200	3.022	0.711
IL-10	4	144/153	0.487 (−0.235–1.210)	1.322	0.186	34.235	3.000	< 0.001	91.237	0.732	0.935
IL-17	9	401/312	1.452 (0.331–2.572)	2.540	0.011	306.597	8.000	< 0.001	97.391	8.950	0.115

*GBS, Guillain Barré Syndrome; df, degrees of freedom; IFN-γ, interferon γ; IL, interleukin; TNF, tumor necrosis factor; TGF, transforming growth factor; CRP, C-reactive protein*.

### Main Associations of GBS With CSF Cytokines

**Figure 4:**
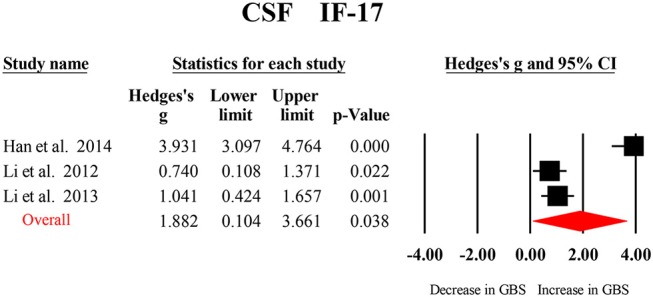
Studies of cerebrospinal fluid IL-17 in Guillain Barré Syndrome. Forest plot displaying random-effects meta-analysis result of the association between IL17 and Guillain Barré Syndrome. The sizes of the squares are proportional to study weights.

Only CSF IL-17 were analyzed in the three studies included in this meta-analysis, and the results from the meta-analysis indicated that CSF IL-17 levels were significantly increased in patients with GBS compared to the healthy controls (Figure 4; Table 2).

**Table 2:** Summary of comparative outcomes for measurements of CSF cytokine levels.

**CSF cytokine**	**No. of studies**	**No. with GBS/controls**	**Main effect**			**Heterogeneity**				**Publication bias**	
			**Hedges g (95% CI)**	***z* score**	***p* value**	**Q statistic**	***df***	***p* value**	**I^**2**^ statistic**	**Egger intercept**	***p* value**
IL-17	3	96/58	1.882 (0.104–3.661)	2.075	0.038	40.286	2.000	<0.001	95.036	28.004	0.117

*GBS, Guillain Barré Syndrome; df, degrees of freedom; IL, interleukin; CSF, cerebrospinal fluid*.

### Investigation of Heterogeneity

Our analyses suggested that all the blood and CSF cytokines had high levels of between-study heterogeneity (Tables 1, 2). Subsequently, we tried to perform sub-group and meta-regression analyses to address whether the relevant categorical and continuous variables would affect the between-study heterogeneity. As shown in the Supplementary Table 1, the data on disease duration, disease severity, and medication status were limited, and therefore, meta-regressions were performed on age, gender, and publication year. Additionally, since most cytokines were not analyzed in less than 10 studies, blood TNF-α was chosen for sub-group and meta-regression analyses.

Meta-regression analyses suggested that age, gender, and publication year did not significantly affect the outcomes of the meta-analysis (*p* > 0.05 in all the analyses).

Sub-group analyses based on sampling source performed for blood TNF-α did not change the impact of heterogeneity (Q = 165.152, df = 8, *p* < 0.001, I^2^ = 95.156), and the significance of the association between elevated serum TNF-α levels and GBS was retained (Hedges g, 1.869; 95% CI, 0.948–2.790; *p* < 0.001). Contrastingly, there was no significant association between plasma TNF-α levels and GBS (Hedges g, 0.660; 95% CI, −0.068–1.388; *p* = 0.076), whereas, a mild reduction was noted in the impact of heterogeneity (Q = 12.465, df = 2, *p* = 0.002, I^2^ = 83.955).

A sensitivity analysis was additionally performed, and the results indicated that the significant association between blood TNF-α and GBS was not influenced by any single study. Furthermore, the Egger test suggested no significant publication bias risk for most cytokines included in the meta-analysis (Tables 1, 2).

## Discussion

Our results demonstrate that blood inflammatory cytokines—TNF-α, IFN-γ, IL-1β, IL-4, IL-6, IL-17, and CRP levels—are significantly increased in the patients with GBS. We found elevated levels of IL-17 in the CSF of patients with GBS. Contrastingly, no significant association was found between blood levels of IL-10 and TGF-β and GBS. For cytokines significantly associated with GBS, the values of ES associated with the results of CSF IL-17, blood TNF-α, IFN-γ, IL-4, IL-17, and CRP were large, and medium to large for blood IL-1β and IL-6. Sensitivity analysis suggested that a significant association between blood TNF-α and GBS was not influenced by any individual study, and no significant publication bias risks were observed for most cytokines included in the meta-analysis. Therefore, the results demonstrate the robustness of the outcomes of the meta-analysis. Although the literature provided inconsistent clinical data for cytokine aberrations in the patients with GBS, this study used the meta-analytic technique to pool the cytokine data and clarify the profile of inflammatory cytokines in GBS patients. Altogether, our results show that Th1-, Th2-, and Th17-related cytokines were highly elevated in patients with GBS, and therefore, provides strong clinical evidence for better understanding the etiology of GBS.

Results from this meta-analysis suggest that blood IL-10 and TGF-β has no significant association with GBS. However, it is unknown whether the non-significant associations were due to the limited number of studies with small sample sizes for these cytokines. For example, a previous meta-analysis by Swardfager et al. published in 2010 could only show the significant increase in the CSF TGF-β levels in patients with Alzheimer's disease (Swardfager et al., 2010), whereas, a recent meta-analysis by Chen et al. included more studies and suggested that CSF TGF-β, MCP-1, and YLK-40 levels were elevated in the patients with Alzheimer's disease (Chen et al., 2018). In addition to the increased circulating levels of inflammatory cytokines in the patients with Alzheimer's diseases, previous meta-analyses also assessed the cytokine levels in other neurodegenerative diseases of the central nervous system, including Parkinson's disease (Qin et al., 2016) and amyotrophic lateral sclerosis (Hu et al., 2017). However, the values of ES associated with the results of cytokines were mostly small to medium, presenting difficulties in the use of cytokines for informed diagnosis, prognosis, and treatment response for Alzheimer's disease, Parkinson's disease, and amyotrophic lateral sclerosis. Contrastingly, our meta-analysis indicated that the ES values associated with the results of cytokines were mostly large. The large ES values for the associations between blood cytokines and GBS are justified, since GBS is an autoimmune disease which damages the peripheral nervous system, suggesting that cytokines are closely associated with the pathophysiology of GBS. In fact, it has been demonstrated that the infiltrating T cell-produced TNF-α has a direct myelinotoxic effect on myelinated fibers which leads to demyelination in nerve cells, and TNF-α can also affect the synthesis of myelin protein and glycolipids (Lisak et al., 1997; Nyati and Prasad, 2014). Furthermore, in an animal model of GBS-experimental autoimmune neuritis (EAN), it has been shown that the severity of clinical signs of EAN was associated with IL-17 accumulation in the sciatic nerve, and administration of IL-17 exacerbated the clinical signs of the acute phase in chronic EAN (Pelidou et al., 2000). The potential role of IL-17 in the pathogenesis of GBS was further supported by the results suggesting that down-regulation of IL-17 in peripheral blood and /or sciatic nerves contributed to the treatment effects of FTY720, atorvastatin and AUY954 on EAN (Wu et al., 2016). Altogether, these results suggest that cytokine aberrations are pathogenic in GBS.

High levels of between-study heterogeneity were found for all the cytokines analyzed in this meta-analysis. We tried to adjust for potential confounders which may explain the between-study heterogeneity with sub-groups and the results of the meta-regression analyses. However, meta-regression analyses based on age, gender, and publication year did not address the between-study heterogeneity. Although the sub-group analyses indicated a sampling source to partially explain the between-study heterogeneity, while the lower heterogeneity in plasma cytokine-based studies may be due to the low power of the test for heterogeneity in meta-analyses with a smaller number of studies. Moreover, we found that serum TNF-α levels we significantly elevated in the patients with GBS, whereas no significant association between plasma TNF-α levels and GBS was observed, suggesting that the difference of sample sources may influence results of cytokine study. However, it is likely that the small number of studies on plasma TNF-α levels in GBS made it difficult to observe statistical significance. Other clinical variables such as disease duration, disease severity, and medication status may contribute to the between-study heterogeneity in the meta-analysis. In fact, data from Deng et al. show increased Th1-related cytokine, IL-12p70, levels in the serum of patients with GBS, and intravenous immunoglobulin therapy down-regulated the IL-12p70 levels into healthy controls (Deng et al., 2008). Similarly, Radhakrishnan et al. demonstrated that intravenous immunoglobulin therapy in patients with GBS reduced the blood TNF-α levels (Radhakrishnan et al., 2004). However, most studies included in this meta-analysis did not reveal the medication status. This limitation prevented us from performing a meta-analysis to investigate the usefulness of cytokines as biomarkers of treatment responses in GBS.

Another limitation of this meta-analysis is that most of the included studies did not provide information on the stage of GBS. Indeed, Nyati et al. showed that in comparison to healthy controls, blood TNF-α and IL-1β levels were significantly increased in patients in the progressive phase of GBS, whereas, TNF-α and IL-1β levels did not show significant differences between GBS patients and healthy individuals at the recovery phase (Nyati et al., 2010). Therefore, it is likely that the between-study heterogeneity found in this meta-analysis was contributed by the different stages of patients across studies. Nevertheless, these results highlight the need for continuous investigations into the cytokine aberrations in patients with GBS and controlling the relevant clinical and methodological variables to better understand the mechanisms underlying the disease. The last limitation of this study is that only English–language articles were included. However, owing to the limited number of non-English records in the literature on the associations between cytokines and GBS, the outcomes of our meta-analysis are unlikely to be significantly influenced by the non-English articles.

In conclusion, this is the first systematic review and meta-analysis to assess the dysfunctions of blood and CSF inflammatory cytokines in patients with GBS. Our analysis indicates that the peripheral blood levels of inflammatory cytokines are highly elevated in patients with GBS. Therefore, the cytokines have potential to be used as biomarkers to inform diagnosis, prognosis, or treatment responses in GBS, and future studies are necessary to validate this hypothesis.

## Author Contributions

YC and QL conceived and designed the study. TS and XC performed the literature search and data analysis. YC drafted the manuscript with critical revisions from TS, XC, SS, and QL.

### Conflict of Interest Statement

The authors declare that the research was conducted in the absence of any commercial or financial relationships that could be construed as a potential conflict of interest.
