# Supplementary material for: Peripheral Blood and Cerebrospinal Fluid Cytokine Levels in Guillain Barré Syndrome: A Systematic Review and Meta-Analysis
Source: Front Neurosci. 2019 Jul 16;13:717. doi: 10.3389/fnins.2019.00717 (PMC6646663; doi:10.3389/fnins.2019.00717)
Supplement: Supplementary file 1 [file Table_1.DOCX]

**Supplementary Table. Characteristics of included studies measuring blood and CSF cytokine concentrations**

| **Study/Year** | **Cytokines Measured** | **Country** | **Samples**  **(Disease/Ctrl)** | **Gender (%Male) (Disease/Ctrl)** | **Mean Age** **(Disease/Ctrl)** | **Sample Source** | **Sample Collect Time** | **Disease status severity/EDSS** | **Diagnosis** | **Assay type** | **Medication** |
| --- | --- | --- | --- | --- | --- | --- | --- | --- | --- | --- | --- |
| Beppu et al. 2015 | TNF-α， IL-1β | Japan | 18/19 | 50/NA | NA | Serum | NA | NA | NA | Bio-Plex cytokine reagent kit | NA |
| Chang et al.2016 | TGF-β, IL-6, | China | 28/15 | 46.4/53.3 | 47.0 /47.0 | serum | NA | Hadden’s criteria 2.21 | Hadden’s criteria | ELISA | NA |
| Créange et al. 1998 | TNF-α, TGF-β | France | 15/10 | NA | NA | plasma | 4.8 d | NA | Clinical and electrophysiological diagnostic criteria | ELISA | IVIg,PE |
| Debnath et al. 2018 | IL-17A | India | 107/111 | NA | 36.28/NA | Plasma | 7.75 d | Mean MRC sum score 16.75 | Hughes Disability scale | flow cytometer | NA |
| Debnath, et al. 2018 | IL-1β, IL-4, IL-6, IL-10, IL-17A, IFN-γ, TNF-α | India | 65/73 | 66.2/69.9 | 35.80 /32.92 | plasma | NA | NA | Hadden’s criteria | Bioplex 200 analyzer | NA |
| Deng et al. 2008 | TNF-α | China | 28/13 | 57.1/46.1 | NA | Serum | NA | Disability score 3.4 | Hughes degree | ELISA | IVIg or NO |
| Doncel-Pérez et al. 2016 | IL-1β, IL-17, TNF-β | Spain | 8/4 | 50/NA | 57/NA | Serum | NA | NA | NA | ELISA | NA |
| Du et al. 2015 | TNF-α, IL-1β | China | 18/20 | NA | NA | blood | NA | NA | Hughes scale | qPCR | IMMULITE 1000 |
| Ethemoglu et al. 2018 | CRP | Turkey | Adults36/33，Children32/30 | NA | 48.5/48.5，5.68/6.45 | serum | NA | NA | Hughes Disability scale | NA | NA |
| Exley et al.1994 | TNF-α, IL-1β, INF-α | UK | 26/23 | NA | NA | plasma | NA | NA | Asbury, A.K. diagnostic criteria | ELISA | PE |
| Han et al. 2014 | IL-17 | China | 51/20 | 43.1/50.0 | 35.3/34.7 | plasma, CSF | 5.43 d | Disability grad 3.43 | motor nerve conduction criteria | ELISA | IVIg |
| Hohnoki et al. 1998 | IFN-γ, IL-4, IL-10, TNF-α | Japan | 14/12 | 50/50 | 44.1/28.3 | serum | NA | NA | ad hoc NINCDS Committee | ELISA | NA |
| Kharwar, et al. 2017 | IL-17 | India | 80/75 | 80.0/58.7 | 30.20/28.12 | Serum | NA | NA | Arthur K criteria | ELISA, mRNA | NA |
| Li et al. 2012 | IL-17 | China | 22/18 | 63.6/61.1 | 41.91/39.11 | plasma CSF | NA | NA | GBS disability scale scores (GDSs) | ELISA | NA |
| Li et al. 2013 | IL-17A, IFN- γ, TNF-α | China | 25/20 | 66.7/66.7 | 39/32 | plasma | NA | NA | Asbury, A.K. diagnostic criteria | ELISA | IVIg |
| Li et al. 2014 | IL-17 | China | 29/20 | 69.0/70.0 | 41.41/40.35 | plasma | NA | NA | motor nerve conduction criteria | ELISA | IVIg or NO |
| Li et al. 2018 | log CRP | China | 101/111 | 64.4/64.0 | 39.69/41.89 | serum | NA | NA | GBS disability scale scores (GDSs) | Automatic biochemical Analyser | NA |
| Li et al. 2018 | CRP | China | 114/120 | 60.5/55.8 | 37.41 /38.37 | blood | NA | NA | GBS disability scale scores | Automatic Biochemical Analyzer | NA |
| Liang et al. 2012 | IFN-γ, IL-17 | China | 29/29 | 62.1/51.7 | 37.38/45.03 | Serum | 3 d | NA | Asbury, A.K. diagnostic criteria | ELISA | NA |
| Nyati et al. 2010 | TNF-α，IL-1β | India | 65/68 | 61.5/64.7 | 40.29/41.90 | Serum | 4.5 d | NA | Hadden’s criteria | ELISA | IVIg |
| Nyati et al. 2011 | IFN-γ, IL-1β, TNF, IL-6, TGF-β, IL-4, IL-10 | India | 65/68 | 61.5/64.7 | 40.29/41.90 | blood | 4.5 d | NA | Hadden’s criteria | ELISA | IVIg |
| Ossege et al. 2000 | TNF-α, TGF-β | Germany | 10/10 | 0/NA | NA | CSF, serum | NA | Clinical score 2.5 | Winer score | ELISA, mRNA | IVIG, IA PP |
| Press et al. 2001 | IL-10, IFN-γ | Sweden | 41/55 | 56.1/NA | 53 | blood | NA | NA | Asbury criteria | ELISA | IvIg |
| Radhakrishnan et al. 2003 | TNF-α | India | 57/25 | NA | 34/NA | Serum | NA | NA | Hadden’s criteria | ELISA | IVIg |
| Radhakrishnan et al. 2004 | TNF-α | India | 41/25 | NA | NA | Serum | NA | Hughes 3.3 | Asbury’s criteria | ELISA | IVIG, PE |
| Sainaghi et al. 2010 | IL-1β | Italy | 9/7 | NA | NA | CSF | NA | NA | Asbury diagnostic criteria | ELISA | NA |
| Sharief et al. 1993 | TNFα | England | 26/14 | 53.8/64.3 | 39 /19 | Serum CSF | NA | 4.000 | Arthur K criteria | ELISA | NA |
| Sindern et al. 1996 | TGF-β | Germany | 12/12 | NA | NA | Serum | 5.2 d | NA | Arthur K criteria | ELISA | IVIg,PE |
| Wang et al. 2012 | IFN-γ, CRP | China | 21/21 | 47.6/52.3 | 32.38/34.68 | Serum | NA | Disability score 2.71±1.15 | Arthur K criteria | ELISA | IVIg, PE, Supportive cure |
| Zhang et al. 2016 | IL-6, TNF-α | China | 86/60 | 44.2/NA | 44.7/NA | Serum | NA | NA | Asbury and Cornblath criteria | ELISA | NA |

**Abbreviations:** GBS, Guillain Barré Syndrome; *df*, degrees of freedom; IFN-γ, interferon γ; IL, interleukin; TNF, tumor necrosis factor; TGF, transforming growth factor; CRP, C-reactive protein. EDSS, [Expanded Disability Status Scale](https://www.baidu.com/link?url=WOHfXDftCrqGRkPkop1CBV1m6PZpef14-ulKqARoJwK5xman0Wt0QVuaPt2Pz9YiMwDXMELyI5S2GoXR5Z8dnJnLrY62wa37FsQaRvunMsm&wd=&eqid=b37ed2d30007eaf0000000035c52fa64); ELISA, Enzyme-Linked ImmunoSorbent Assay.
